# A Low Complexity Sensing Algorithm for Non-Sparse Wideband Spectrum

**DOI:** 10.3390/s22166295

**Published:** 2022-08-21

**Authors:** Shiyu Ren, Wantong Chen, Hailong Wu, Dongxia Li, Zhongwei Hu

**Affiliations:** School of Electronic Information and Automation, Civil Aviation University of China, Tianjin 300300, China

**Keywords:** wideband spectrum sensing, non-sparse spectrum, folded time-frequency spectrum, time-frequency subband classification

## Abstract

The vast majority of existing sub-Nyquist sampling wideband spectrum sensing (WSS) methods default to a sparse spectrum. However, research data suggests that in the near future, the wideband spectrum will no longer be sparse. This article proposes a sub-Nyquist sampling WSS algorithm that can adapt well to non-sparse spectrum scenarios. The algorithm continues to implement the idea of our previously proposed “no reconstruction (NoR) of spectrum” algorithm, thus having low computational complexity. The new one is actually an advanced version of the NoR algorithm, so it is called AdNoR. The key to its advancement lies in the establishment of a folded time-frequency (TF) spectrum model with the same special structure as in the fold spectrum model of the NoR algorithm. For this purpose, we have designed a comprehensive sampling technique which consists of multicoset sampling, digital fractional delay, and TF transform. It is verified by simulation that the AdNoR algorithm maintains a good sensing performance with low computational complexity in the non-sparse scenario.

## 1. Introduction

Cognitive radio (CR) is a promising technology that will enable future wireless systems to make efficient use of the spectrum resource. A key component of CR is spectrum sensing. As future CR networks are planned to operate over a wide frequency range, wideband spectrum sensing (WSS), which can quickly and reliably detect idle spectrum in a wide band, is essential. Early Nyquist sampling-based WSS methods faced hardware implementation challenges because high-speed analog-to-digital converters (ADCs) are energy-intensive and too costly for practical systems. Because compressed sensing (CS) was first applied to wideband spectrum sensing (WSS) [1], sub-Nyquist sampling-based WSS methods have received a lot of attention from experts, and the development momentum has been unstoppable. Some fairly classical CS-based methods, such as those non-convex optimization-based [2] and greedy pursuit-based [3], although they are good solutions to the problem of high Nyquist sampling rate for WSS, they require signal recovery, which brings a large computational burden. To overcome the disadvantage of high computational complexity of the CS-based method, some later works propose reconstructing the power spectrum [4] or covariance matrix [5] of the wideband signal from sub-Nyquist samples obtained by the multichannel sampling scheme. This class of methods are referred to as compressive covariance sensing (CCS)-based methods, and such methods have the additional advantage of not requiring a sparse prior [5]. Then, based on the idea of the CCS-based method, we successively proposed a series of multichannel sampling scheme based methods [6,7,8,9]. It is evident that we were forward looking in choosing our research direction in the first place, because later in [10], a comparative study concludes that the CCS-based method provides a more competitive alternative for reliable WSS.

Recent research on WSS methods takes more into account the practical applicability, i.e., the algorithms are designed with more emphasis on incorporating practical application scenarios. For example, among some articles that study WSS methods, there are those that consider the situation of severe wireless channel fading [11], the scenario of non-contiguous wideband spectrum [12], and most interestingly, the scenario of electronic warfare applications [13]. Yet there is a class of application scenarios for WSS that is rarely addressed, namely, non-sparse wideband spectrum. Actually, there’s a reason for that. The Institute of Wireless Broadband Mobile Communication of Beijing Jiaotong University has measured the spectrum utilization in Beijing and found that the spectrum utilization in the 698∼806 MHz is 21.29%, the spectrum utilization in the 850∼970 MHz is about 2.33%, and the spectrum utilization in the 1000∼2000 MHz is less than 1% [14]. Another example is that in aviation communication systems, the spectrum utilization of the air-ground communication band is less than 12.5%, and the utilization of the Very High Frequency (VHF) band (117.95∼137 MHz), which is used for air traffic control, is only 5% [15]. It can be seen that the current wireless broadband communication spectrum is sparse in general. However, over the next decade, mobile data traffic will grow approximately 1000 times, and in future networks, it is expected that wireless systems should achieve a significant increase of at least 10× to 1000× in capacity and spectral efficiency [16]. The above predictions imply that in the near future, wideband spectrum will no longer be sparse. In fact, even now, the increase in the number of electronic surveillance, radar and communication devices, and the proliferation of wideband non-smooth signals, such as Gaussian pulses, frequency stepping signals and linear FM signals make the received signal at a certain sensing time no longer sparse, so that the sparsity dependent sub-Nyquist WSS method will not be applied [17].

Despite the imminence of research on WSS in non-sparse scenarios, however, few works have studied it. Some scholars once considered “what if spectrum is not sparse?”, then a collaborative non-sparsity protection scheme that can efficiently identify spectrum sensing failures was proposed [18]. Several others have proposed another method for detecting whether the reconstruction process is unsuccessful due to the non-sparse spectrum in modulated wideband converter based WSS [19]. In [20], the authors claimed that they have provided the first solution to the problem of WSS in the case of non-sparse scenario, yet the wideband spectrum to be detected in their simulation experiments contains only 16 subbands. The authors in [21] proposed a sparsity independent sub-Nyquist WSS method on real-time TV white space. However, there are some shortcomings. First, prior information on the number of channels and input spectrum utilization is needed; second, a complex process containing three main blocks—signal permutation and filtering, spectrum estimation and multi-channel joint detection, and exaggeratingly, the first block is divided into another three additional steps; third, computational complexity is not truly reflected because of incomplete calculation of the simulation runtime.

To enable simple and efficient sub-Nyquist WSS in the non-sparse scenario, we propose an algorithm with low computational complexity that does not require spectrum reconstruction. The key, or contribution, of our algorithm is as follows.

(1) Algorithm Design: We extend our previous non-reconstruction (NoR) algorithm idea [7] to propose an advanced version, namely AdNoR, due to the fact that the original version requires the spectrum to be sparse. The AdNoR extends the sensing domain from the frequency domain to the time-frequency (TF) domain, which also avoids spectrum reconstruction but is free from sparsity constraints. We establish a folded TF spectrum model that satisfies the special structure [7]. Based on which, we first pick out the active aliased TF sub-channel that contains the active signal and then identify the exact location of the active TF subband in the active aliased TF sub-channel.

(2) Modeling: In order to build the folded TF spectrum with particular structure, we design a comprehensive sampling technique based on the multicoset sampling setting. This technique is composed of multicoset sampling, digital fractional delay (DFD), and TF transform. The technical difficulty lies in the incorporation of DFD, which is a functional module that is back-performed after theoretical reasoning.

(3) Simulation verification and analysis: Because CCS-based methods are not subject to sparse restrictions, CCS-based methods are generally better adapted to non-sparse scenarios than CS-based methods. So, we first choose an excellent representative of CCS-based methods, the ADP algorithm [9], which has just been proposed in the last year and motivated by practical applications, as a comparison algorithm. Furthermore, we choose two additional algorithms for comparison. One is the orthogonal matching pursuit (OMP) algorithm [3], a representative of CS-based methods, and the other is the NoR algorithm. Experimental simulations and computational complexity analysis prove that when the spectrum is not sparse, the AdNoR not only has much better detection performance than the other three algorithms but also has a low computational complexity comparable to that of the NoR and ADP algorithms.

**Figure 1:**
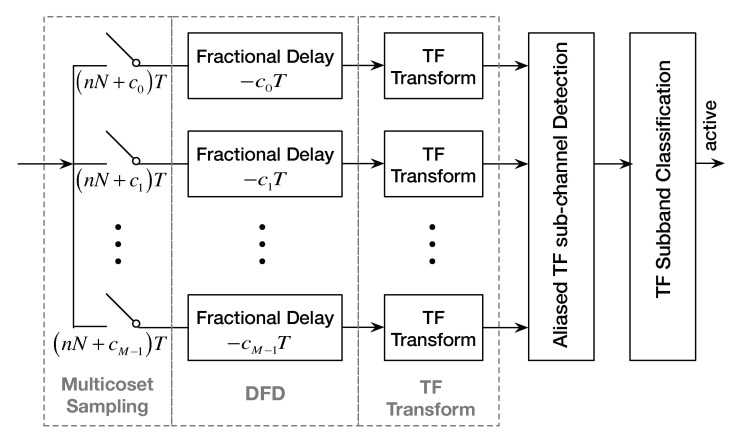
The diagram of the AdNoR algorithm.

In summary, the flow of the AdNoR algorithm is shown in Figure 1. First, a folded TF spectrum model is established. This step is implemented by three functional modules: multicoset sampling, DFD, and TF transform. This section will be covered in detail in Section 2. Next is the algorithm design part. Based on the folded TF spectrum model, we select the active aliased TF sub-channel who contains active signal with the help of module “Aliased TF Sub-channel Detection”. Then, we identify the exact location of active TF subband in the active aliased TF sub-channel by module “TF Subband Classification”. This part will be described in detail in Section 3. Simulation results are presented and analyzed in Section 4. We finally make the conclusion in Section 5.

## 2. Folded Time-Frequency Spectrum Model

Suppose that a wideband signal $x\left(t\right)$ with Nyquist rate 11TT is to be sensed. It consists of *U* consecutive non-overlapping subbands.

### 2.1. Multicoset Sampling and Digital Fractional Delay

As shown in Figure 1, we use the multicoset sampling scheme to acquire the signal $x\left(t\right)$. This sampling scheme uses *M* parallel A/D cosets (or branches) to sample the signal uniformly at a declined rate 11NTNT, where *N* is the down-sampling factor. Thus, sub-Nyquist sampling can be satisfied by M<N. For the *i*-th coset, the sampling offset is set to $c_{i}T\left(c_{i}\in Z\right)$, and $0⩽c_{0}<c_{1}<\cdots <c_{M-1}⩽N-1$. Then, the sub-Nyquist sampled signal sequence of the *i*-th coset is obtained:

(1) $${\tilde{x}}_{i}\left[n\right]=x\left(\left(nN+c_{i}\right)T\right),i=0,1,\cdots ,M-1$$

Equation (1) represents the function of module “Multicoset Sampling” in Figure 1. Furthermore, $\left(nN+c_{i}\right)T,i=0,1,\cdots ,M-1$ in the figure represents the sampling time point of the *i*-th coset.

We assume that $X\left(\omega \right)$ is the Fourier transform of $x\left(t\right)$ and is band-limited to 0,2π2πTT. So, we can write the Discrete Time Fourier Transform (DTFT) of ${\tilde{x}}_{i}\left[n\right]$ as [22]:

(2) X˜iejωNT=1NT∑l=0N−1e−jciTω−2πl2πlNTNTXω−2πl2πlNTNT

After multicoset sampling, we apply a digital fractional delay (DFD) before a time-frequency (TF) transform. A reversed digital fractional delay of $-c_{i}T$ to ${\tilde{x}}_{i}\left[n\right]$ yields $y_{i}\left[n\right]$. Furthermore, $y_{i}\left[n\right],i=0,1,\cdots ,M-1$ is the output of the “DFD” module in Figure 1. In fact, the function of the “DFD” module is essentially to delay the samples for a corresponding period of time before feeding them into the “TF Transform” module. In order to relate the subsequent derivation of the formulae of the “TF Transform” module, we calculate the DTFT of $y_{i}\left[n\right]$ as [22].

(3) YiejωNT=1NT∑l=0N−1ej2πlci2πlciNNXω−2πl2πlNTNT

### 2.2. Time-Frequency Transform

(1) TF Representation: We consider a Gabor-based time-frequency transform with the TF atom of [23]

(4) $$g_{p,k}\left(t\right)=g\left(t-p\tau_{0}\right)e^{j2\pi k\xi_{0}t}$$

where $g\left(t\right)$ denotes the window function. It is assumed to be normalized, ${∥g∥}_{2}=1$, basically band-limited to ω∈0,2π2πNTNT, and with the temporal support of $t\in \left[0,NLT\right)$. The parameters $\tau_{0}$ and $\xi_{0}$ define the discrete TF lattice $\left(\tau ,\xi \right)\in \left\{\left(p\tau_{0},k\xi_{0}\right)\left|\left(p,k\right)\in Z^{2}\right.\right\}$ of the TF transform. For analytical convenience, we let $\tau_{0}=PNT$ for some integer *P* and ξ0=11FNTFNT for the integer F=UUNN. Then, TF representations (or coefficients) of $x\left(t\right)$ can be given by:

(5) sp,k=xt,gp,kt=∫−∞∞xtg*t−pτ0e−j2πkt−j2πktNFTNFTdt=12π∫02πXω+2πk2πkNFTNFTG*ωe−jωpPNTdω

where the last line is derived from the Plancherel formula. Based on the band-limited assumption of $g\left(t\right)$, this can be well approximated by:

(6) sp,k≈12π∫02π2πNTNTXω+2πk2πkNFTNFTG*ωe−jωpPNTdω

(2) Sub-Nyquist TF Representation: Consider now the sub-Nyquist TF representation for a single coset signal $y_{i}\left[n\right]$. Because $g\left(t\right)$ is basically bandlimited, the discrete time sampled atoms can be used:

(7) gp,kn=gn−pPej2πknnFF

Then, similar to the derivation of (5), the sub-Nyquist TF coefficients can be calculated as:

(8) qp,ki=yin,gp,kn=∑n=pPpP+L−1ying*n−pPe−j2πkn−j2πknFF=12π∫02π2πNTNTYiω+2πk2πkNFTNFTG*ωe−jωpPNTdω

Let $G_{d}\left(e^{j\omega NT}\right)$ denote the DTFT of $g\left[n\right]$, then we have:

(9) GdejωNT=1NT∑k=−∞∞Gω−2πk2πkNTNT≈1NTGω,0⩽ω<2π2πNTNT

where the approximation is based on the basically band-limited assumption. From (9), Equation (8) can be rewritten as:

(10) qp,ki=NT2π∫02πNTYiejωNT+2πk2πkFFGd*ejωNTe−jωpPNTdω

Furthermore, from (3), we can also have:

(11) YiejωNT+2πk2πkFF=1NT∑l=0N−1ej2πlcij2πlciNNXω+2πk2πkNFTNFT−2πl2πlNTNT

Substitute (11) into (10), we obtain:

(12) qp,ki≈12πNT∑l=0N−1∫02π2πNTNTej2πlcij2πlciNN·Xω+2πk2πkNFTNFT−2πl2πlNTNTG*ωe−jωpPNTdω≈1NT∑l=0N−1ej2πlcij2πlciNNsp,k+lF

From (12), the sub-Nyquist TF representations can be rewritten as:

(13) $$\begin{array}{c} \underset{q_{p,k}}{\underset{︸}{\left[\begin{array}{c} q_{p,k}^{\left(0\right)}\\ q_{p,k}^{\left(1\right)}\\ \vdots \\ q_{p,k}^{\left(M-1\right)} \end{array}\right]}}=\frac{1}{NT}\underset{C}{\underset{︸}{\left[\begin{array}{cccc} e^{j\frac{2\pi }{N}c_{0}0} & e^{j\frac{2\pi }{N}c_{0}1} & \cdots & e^{j\frac{2\pi }{N}c_{0}\left(N-1\right)}\\ e^{j\frac{2\pi }{N}c_{1}0} & e^{j\frac{2\pi }{N}c_{1}1} & \cdots & e^{j\frac{2\pi }{N}c_{1}\left(N-1\right)}\\ \vdots & \vdots & \vdots & \vdots \\ e^{j\frac{2\pi }{N}c_{M-1}0} & e^{j\frac{2\pi }{N}c_{M-1}1} & \cdots & e^{j\frac{2\pi }{N}c_{M-1}\left(N-1\right)} \end{array}\right]}}\cdot \underset{s_{p,k}}{\underset{︸}{\left[\begin{array}{c} s_{p,k+F0}\\ s_{p,k+F1}\\ \vdots \\ s_{p,k+F\left(N-1\right)} \end{array}\right]}}\\ k=0,1,\cdots ,F-1 \end{array}$$

Furthermore, Equation (13) is the TF representations of the output of the “TF Transform” module in Figure 1.

### 2.3. Model Display

**Figure 2:**
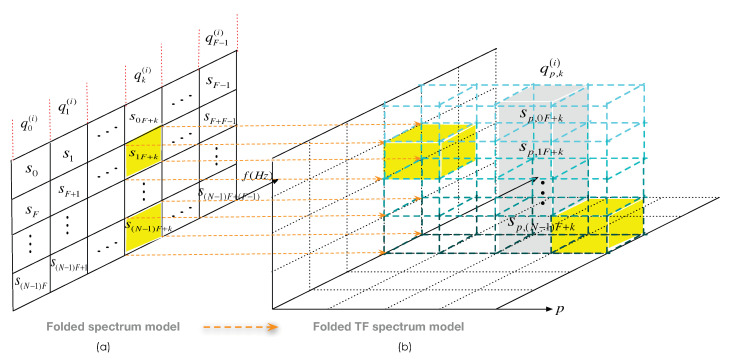
(**a**) The virtual display of the folded spectrum of NoR algorithm. (**b**) The virtual display of the folded TF spectrum of AdNoR algorithm.

Figure 2b is the virtual model display of (13), i.e., the folded time-frequency spectrum model. Furthermore, Figure 2a shows the folded frequency spectrum obtained in the article of our proposed NoR algorithm. It is easy to see that the folded TF spectrum is essentially an extension of the folded frequency spectrum in the time domain.

In the figure, we use the transform representation of each subband to represent itself. In Figure 2b, there are a total of NFP (i.e., UP) TF subbands, each of which is graphically represented by a small box. For example, one of the small boxes $s_{p,nF+k}$ is essentially the $\left(p,nF+k\right)$-th TF subband in the Nyquist TF lattice. In addition, there are a total of FP aliased TF sub-channels, each represented by a cubic column. Furthermore, the cubic column marked in gray $q_{p,k}^{\left(i\right)}$ is essentially the $\left(p,k\right)$-th aliased TF sub-channel in the sub-Nyquist TF lattice obtained at the *i*-th sampling coset. In Figure 2a, each cell $s_{nF+k}$ represents the $\left(nF+k\right)$-th of the *U* subbands. Furthermore, each planar column $q_{k}^{\left(i\right)}$ represents the *k*-th of the *F* aliased sub-channels obtained from the *i*-th sampling coset.

Moreover, the cell or box marked in yellow represents that this subband or TF subband is occupied (active). Furthermore, we determine that the aliased sub-channel (or aliased TF sub-channel) containing any active subband (or TF subband) is active and vice versa.

## 3. AdNoR Algorithm

In this section, we introduce a special structure of the folded TF spectrum named ADFS. Based on the ADFS structure, we first identify the active aliased TF sub-channels and then find that dominant subband for each of them.

### 3.1. ADFS Structure

**Definition** **1.** *Approximate Disjoint Folded Subband (ADFS). We say that the folded TF spectrum has an ADFS structure if each active aliased TF sub-channel is dominated only by a single active TF subband, such that:*

(14) $$q_{p,k}^{\left(i\right)}\approx \frac{1}{NT}e^{j\frac{2\pi }{N}c_{i}l}s_{p,k+Fl}\;$$

*This structure is reflected in Figure 2b that there are no two or more small yellow boxes in a cubic column at the same time.*

The idea of our previously proposed NoR algorithm is based on the ADFS structure. To ensure that ADFS holds, the NoR algorithm needs to satisfy the restriction that the spectrum must be sparse, i.e., D≪U, where *D* represents the number of active subbands. The AdNoR algorithm is based on the same idea as the NoR algorithm but without the restriction D≪U. The reason is as follows. Based on the fact that the vast majority of signals in wireless environments are non-smooth, even if the wideband spectrum is not sparse, the sparsity condition is easily satisfied when expanded to the time-frequency domain [17]. That is, no restriction of D≪U is required and ADFS still holds with high probability.

We illustrate the above features graphically in Figure 2. As in the folded spectrum model of Figure 2a, a certain aliased sub-channel contains two active subbands, i.e., ADFS is not valid. However, in Figure 2b, when these two active subbands are extended to the TF domain, they are in different aliased TF sub-channels, i.e., ADFS holds in the folded TF spectrum.

### 3.2. Aliased TF Sub-Channel Detection

Based on ADFS structure, the aliased TF sub-channel for each sampling coset should have a similar magnitude, that is

(15) $${∥q_{p,k}^{\left(0\right)}∥}_{2}\approx {∥q_{p,k}^{\left(1\right)}∥}_{2}\approx \cdots \approx {∥q_{p,k}^{\left(M-1\right)}∥}_{2}$$

The test statistic of the aliased TF sub-channel detection is set to $E\left[q_{p,k}\right]=\sum_{i=0}^{M-1}{∥q_{p,k}^{\left(i\right)}∥}_{2}$. Suppose that the active TF subband signals are independent and zero-mean and here is additive white Gaussian noise with power spectrum density $\sigma^{2}$. Next, a threshold value θ should be chosen to achieve a constant false alarm rate. Because the strategy for calculating the threshold value θ in the AdNoR algorithm is the same as that in the NoR algorithm, it is not repeated here. When $E\left[q_{p,k}\right]>\theta$, we save the index of the active TF sub-channel $\left(p,k\right)$ to the set Ω, which will be used in the next subband classification.

### 3.3. TF Subband Classification

Once an aliased TF sub-channel is identified as active, it needs to be assigned to a TF subband. We can consider this as a classification task. The optimal classification is accomplished under the Gaussian noise assumption by maximizing the absolute inner product between $q_{p,k}$ and the phase vector $\rho \left(l\right)={\left[e^{-j2\pi c_{0}\frac{l}{N}},e^{-j2\pi c_{1}\frac{l}{N}},\cdots ,e^{-j2\pi c_{M-1}\frac{l}{N}}\right]}^{T}$ for the $\left(p,lF+k\right)$-th TF subband:

(16) $$\begin{array}{c} {\hat{l}}_{p,k}=\arg\max_{l}{\left|\sum_{i=0}^{M-1}q_{p,k}^{\left(i\right)}e^{-j2\pi c_{i}\frac{l}{N}}\right|}^{2},k\in \Omega ,l=0,1,\cdots ,N-1 \end{array}$$

Thus, the $\left(p,{\hat{l}}_{p,k}F+k\right)$-th TF subband is allocated as the active one in the corresponding $\left(p,k\right)$-th active TF aliased sub-channel. So far, we can obtain that the $\left({\hat{l}}_{p,k}F+k\right)$-th subband is occupied in the original non-sparse wideband spectrum. In summary, the flow of the AdNoR algorithm is shown in Algorithm 1.
**Algorithm 1** AdNoR Decoder1: **for** each aliased TF sub-channel **do**2:    Calculate $E\left[q_{p,k}\right]=\sum_{i=0}^{M-1}{∥q_{p,k}^{\left(i\right)}∥}_{2}$ as the test statistic.3:   **if** $E\left[q_{p,k}\right]>\theta$ **then**4:     the $\left(p,k\right)$-th aliased TF sub-channel is active;5:     **for** each TF subband who folds into the $\left(p,k\right)$-th aliased TF sub-channel **do**6:      Calculate $a\left(l\right)={\left|\sum_{i=0}^{M-1}q_{p,k}^{\left(i\right)}e^{-j2\pi c_{i}\frac{l}{N}}\right|}^{2}$.7:     **end for**8:     Choose ${\hat{l}}_{p,k}=\arg\max_{l}\left[a\left(l\right)\right]$.9:     the $\left({\hat{l}}_{p,k}F+k\right)$-th subband is active.10:  **end if**11: **end for**


### 3.4. Computational Complexity Analysis

We next analyze the computational complexity of the four algorithms: ADP, OMP, NoR, and AdNoR. The computational complexity is measured by the number of complex float point operations [24]. To create a fair comparison, we select parameters to ensure that the four algorithms have the same number of monitored frequency subbands, compression ratio, and total amount of samples.

For ADP algorithm, its computational complexity is [9]

(17) $$CC_{ADP}=O\left(2FK+\eta_{1}FNM+\left(\eta -\eta_{1}\right)FNM^{2}\right)$$

In the above equation, *K* is the number of training data used in least squares support vector machine (LS-SVM) based sub-channel detection scheme in the ADP. Furthermore, $\eta \;=\;1-{\left(1-\frac{D}{U}\right)}^{N}$, $\eta_{1}=C_{N}^{1}\cdot \frac{D}{U}{\left(1-\frac{D}{U}\right)}^{N-1}$.

For the OMP algorithm, its computational complexity is [9]

(18) $$CC_{OMP}=O\left(DMNF^{2}+\frac{3}{2}D\left(D+1\right)MF+\frac{1}{3}D\left(D+1\right)\left(2D+1\right)\right)$$

For the NoR algorithm, its computational complexity is [9]

(19) $$CC_{NoR}=O\left(FM+DNM\right)$$

The first part of the above equation $O\left(FM\right)$ is the computational complexity of aliased sub-channel detection, whereas the second part $O\left(DNM\right)$ is the computational complexity of the subband classification.

For the AdNoR algorithm, the extra computational effort compared to the NoR algorithm is in the aliased sub-channel detection part. It needs to detect $\left(P-1\right)F$ more aliased sub-channels than the NoR. As for the subband classification part, its computational complexity is related to the number of active subbands *D*, the number of sampling cosets *M* and the down-sampling factor *N*, and these three parameters are set the same in both algorithms. Therefore, the computational complexity of AdNoR is

(20) $$CC_{AdNoR}=O\left(PFM+DNM\right)$$

Due to the uncertainty of *D* in the non-sparse wideband spectrum scenario, it is not convenient to compare the computational complexity of the four algorithms theoretically, so simulation analysis is used to compare them. The details of the comparison are described in the next chapter.

## 4. Simulation

To verify the excellent performance of AdNoR, we establish WSS experiments and compare the performances of ADP, OMP, NoR, and AdNoR algorithms. A wideband divided into U=FN=360 subbands is used, with each subband having a width of 4 MHz. Furthermore, on each subband, there can be at most one primary user that sends data. QPSK symbols are transmitted. For AdNoR, the Gabor transform chooses a Gaussian window with a window length *P*, and *P* is also the number of Gabor coefficients in time.

**Figure 3:**
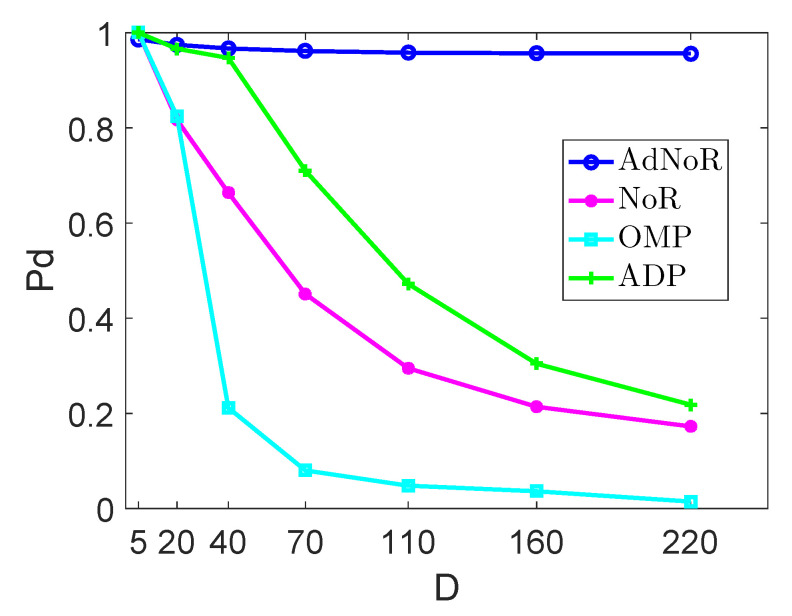
Detection Probabilities of the four sensing algorithms with different number of active subbands D=5,20,40,70,110,160,220, and with U=360,N=8,N/M=2,P=180,SNR=10 dB.

**Figure 4:**
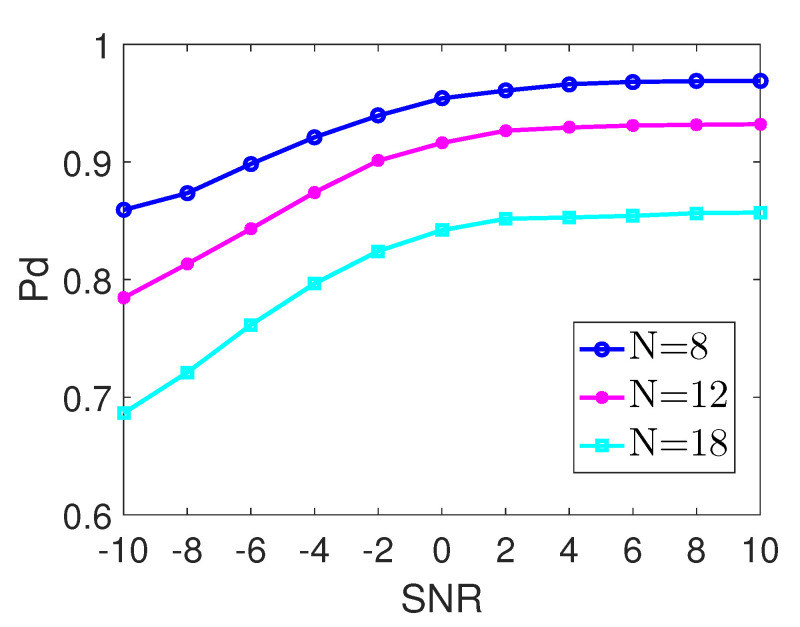
Detection Probabilities of the AdNoR algorithm with different down-sampling factors N=8,12,18, and with U=360,N/M=2,P=180,D=50.

**Figure 5:**
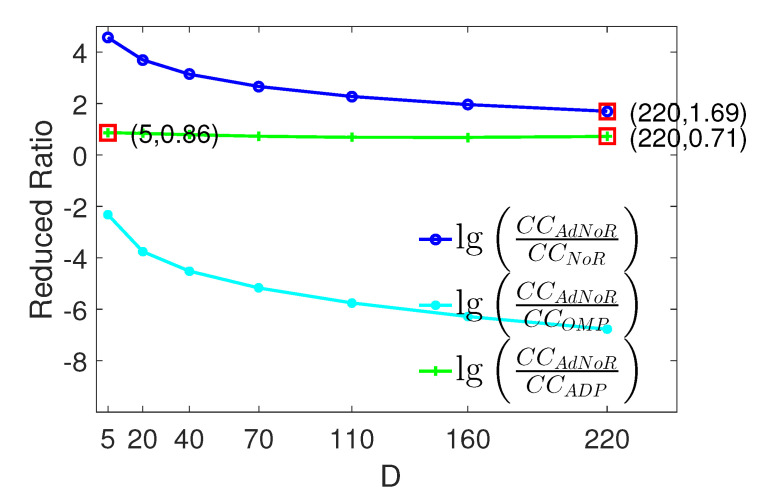
Reduced Ratio of computational complexity of AdNoR against NoR, OMP, and ADP, respectively, with parameters consistent with the first experiment.

(1) Sensing Performance versus Number of Active Subbands *D*: In the first experiment, we set D=5,20,40,70,110,160,220, and the down-sampling factor N=8, the compression ratio NNMM=2, the number of Gabor coefficients in time P=180, the false alarm probability $P_{\mathrm{fa}}\;=\;0.01$, and SNR =10 dB. The number of training data used in the LS-SVM based scheme in ADP is set as K=150. For OMP, the appropriate measurement matrix is chosen to obtain the sub-Nyquist samples based on compression ratio NNMM=2 and total number of subbands *U* to ensure fairness, whereas for *N*,*F* are independent of OMP. The sensing performances are presented in Figure 3. We can see that the advantage of the detection performance of AdNoR over the other three algorithms becomes more obvious as *D* increases. For OMP and NoR, the reason is that they are predicated on spectral sparsity. For ADP, although it is not limited by sparsity, its detection performance degrades rapidly when D>70, i.e., roughly channel occupancy $\frac{D}{U}>20\%$.

(2) Sensing Performance versus Down-sampling Factor *N*: In the second experiment, we set N=8,12,18, NNMM=2, D=50, P=180, and $P_{\mathrm{fa}}\;=\;0.01$. In this experiment, we discuss the effect of *N* on the performance of AdNoR. In Figure 4, we can see that the AdNoR algorithm is significantly affected by *N*, and its performance is decreasing as *N* increases. The reason is as follows: *N*, the down-sampling factor, is also the spectrum folding factor (or degree), and it is easy to understand that as the folding degree increases, the probability of the ADFS structure being satisfied decreases [7], which will directly affect the accuracy of subband classification.

(3) Computational Complexity Comparison: Figure 5 shows a comparison of the computational complexity of the four algorithms drawn from the parameters of the first experiment and Equations (17)–(20). We can see that with reasonable parameter settings, there exists $CC_{NoR}<CC_{ADP}<CC_{AdNoR}<CC_{OMP}$. The computational complexity of AdNoR is slightly greater than that of ADP, with a difference of less than an order of magnitude. Furthermore, as *D* increases, the computational complexity of AdNoR becomes closer to that of the NoR algorithm, whereas the ratio of the complexity of OMP to that of the other three algorithms becomes larger and larger.

(4) Comprehensive Evaluation of the AdNoR Algorithm: Considering Figure 3 and Figure 5 together, when D=220, the difference between the computational complexity of the AdNoR algorithm and that of both the NoR and ADP is about one order of magnitude, whereas its detection probability is much higher than that of the other three algorithms. It can be seen that when *D* is large, i.e., the spectrum is not sparse, the AdNoR algorithm not only has much better detection performance than the other three algorithms but also has a low computational complexity comparable to that of both the NoR and ADP algorithms. Furthermore, the large influence of *N* on the AdNoR algorithm, as reflected in Figure 4, is not a problem. This is because a larger *N* means that the number *M* of sampling cosets required is also higher, i.e., the hardware cost and energy consumption will be higher, which is against the principle of practical applications. Therefore, for the AdNoR algorithm, excellent detection performance can be obtained by choosing the *N* value that matches the actual application scenario. In summary, our AdNoR algorithm is very suitable for the non-sparse scenario.

## 5. Conclusions

In this article, we have proposed a computationally efficient sub-Nyquist rate WSS algorithm in non-sparse scenario without spectrum reconstruction. The AdNoR algorithm performs sensing on the aliased TF spectrum with the ADFS structure obtained by a comprehensive sampling technique. The sampling technique we designed consists of multicoset sampling, digital fractional delay, and TF transform. After simulation performance verification and computational complexity analysis, we make a comprehensive evaluation of the AdNoR algorithm, which is very suitable for the non-sparse scenario.

## Data Availability

Not applicable.
